# Electromechanical wave imaging vs electrocardiographic imaging: a direct comparison of non-invasive ventricular activation mapping modalities

**DOI:** 10.1007/s10840-025-02156-y

**Published:** 2025-11-24

**Authors:** Johanna B. Tonko, Melina Tourni, Aikaterini Afentouli, Joseph Hansen-Shearer, Biao Huang, Mengxing Tang, Anthony Chow, Elisa Konofagou, Pier D. Lambiase

**Affiliations:** 1 Institute for Cardiovascular Science University College London, 5 University Street, London, UK; 2 St. Bartholomew S Hospital, Bart s NHS Health Trust, London, UK; 3 Department of Biomedical Engineering, Columbia University New York, New York, NY 10001 USA; 4 Department of Electrical and Computer Engineering, National Technical University of Athens, Athens, Greece; 5 Ultrasound Laboratory for Imaging and Sensing, Department of Bioengineering, Imperial College London, London, UK; 6 Department of Radiology, Columbia University, New York, NY 10001 USA; 7 Department of Neurological Surgery, Columbia University, New York, NY 10001 USA

**Keywords:** Electromechanical wave imaging, Electrocardiographic imaging, Non-invasive mapping, Ventricular arrhythmia, Catheter ablation, Transmural mapping

## Abstract

**Background:**

Precise non-invasive identification of the site of origin (SoO) of ventricular arrhythmias (VA) could inform ablation strategies.

**Objective:**

To compare spatial accuracy of ultrasound-based electromechanical wave imaging (EWI) and ECG imaging (ECGI) to estimate the anatomical and axial (endo- vs epicardial) SoO of focal VA or pace maps employing contact mapping as gold standard.

**Methods:**

Patients awaiting a catheter ablation procedure underwent preprocedural EWI and ECGI to non-invasively map the SoO of VE/VT or RV and LV pacing sites. A commercial CT-ECGI system was used to reconstruct epicardial activation maps. Unipolar EGM morphology and slew rate were employed to estimate axial SoO. EWI was performed using high frame rate (2000fps) transthoracic echocardiography with simultaneous ECG. Contact mapping and pacing sites were used as gold standard to define SoOs.

**Results:**

Thirty-three patients with 36 maps in total were included, 24 patients for VE/VT activation and 9 for pace-mapping. ECGI correctly identified the segmental VA-SoO/pacing-site in 28/36 maps (77.8%) compared to 28/35 by EWI (80%, p = ns). Erroneous annotations in ECGI related to septal and papillary muscle foci, whereas EWI mostly misannotated in outflow tract and RV SoOs. One patient had insufficient VEs to allow EWI VA mapping. Reconstructed unipolar EGM features of ECGI maps did not reliably differentiate endo- from epicardial SoO. Direct transmural mapping with EWI correctly identified the “transmural” SoO in 27/35 (77.1%).

**Conclusion:**

Both EWI and ECGI localized the anatomical SoO in the majority of cases with site-specific advantages but also shortcomings for both modalities. EWI determines the transmural SoO which cannot be reliably localized using ECGI. ECGI has the advantage of providing mapping of multifocal and/or infrequent VA by offering panoramic single beat mapping.

**Graphical abstract:**

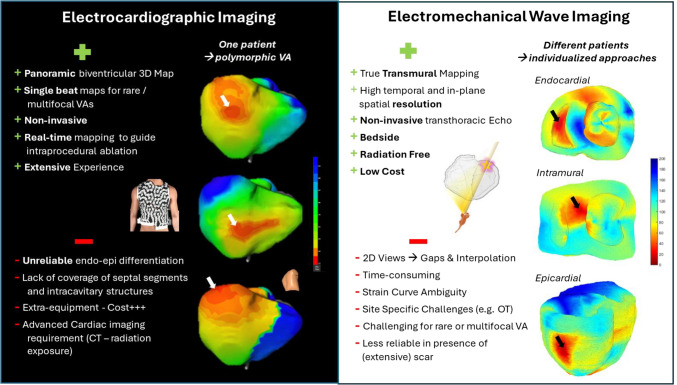

**Supplementary Information:**

The online version contains supplementary material available at 10.1007/s10840-025-02156-y.

## Introduction

Accurate non-invasive mapping of ventricular arrhythmias (VA) would be a valuable diagnostic tool for procedural planning and risk stratification of percutaneous catheter ablation. Developing more reliable non-invasive mapping methods has become even more important in view of novel non-invasive ablation technology (e.g. stereotactic radiotherapy (SABR) [1] and also high-intensity focused ultrasound [2]) that may enable treatment of medically unfit patients for invasive procedures and pave the way for fully non-invasive procedural planning and delivery. While numerous prospective studies are currently ongoing to assess efficacy and safety of SABR [1], there is a comparative lack in innovative and accurate mapping strategies to guide it. Electrocardiographic imaging (ECGI) combining multi-electrode body-surfacevests with anatomical imaging is the only established non-invasive method to generate 3D cardiac activation maps. Its use for VA mapping has shown moderate to good spatial accuracy however with the important short-coming of not providing reliable endo- vs epicardial differentiation [3, 4]. Conversely, electromechanical wave imaging (EWI) is an emerging echocardiography-based modality that provides a low cost, bedside mapping alternative, and we have recently reported on its ability to offer true transmural mapping for VA distinguishing itself from alternative mapping approaches [5].

In this study, we aimed to directly compare the spatial accuracy of the investigational EWI against the established ECGI mapping with anatomical CT to non-invasively estimate the anatomical and transmural (endo- vs epicardial) site of origin (SoO) in a mixed cohort of scar-related and idiopathic ventricular arrhythmias (VA) employing invasive electro-anatomical contact mapping or pacing site as ground truth.

## Methodology

Patients awaiting an elective catheter ablation procedure for ventricular ectopy (VE) or tachycardia (VT) and pre-procedural cardiac MRI to establish presence of structural heart disease and scar pattern were prospectively recruited. All aetiologies were considered.

Included patients underwent a high-frame rate transthoracic echocardiography for EWI and a non-invasive ECGI with anatomical CT using the CardioInsight™ (Medtronic, Cincinnati, US) 3D mapping system in an outpatient or ward-based setting followed by percutaneous catheter ablation with 3D electro-anatomical mapping to serve as ground truth for the accuracy of the non-invasive modalities. In patients without spontaneous arrhythmia at the time of the non-invasive scan/recording (this included all patients with suspected re-entrant VT) but a transvenous device, right endocardial and/or left epicardial ventricular pace maps via the intracardiac device were acquired as surrogate for “focal” sites of origins and lead tip position on CT employed as ground truth. Due to the logistics and set-up of the EWI and ECGI mapping outside of an area that would have offered appropriate monitoring and resuscitation equipment, no programmed ventricular stimulation for VT induction could be performed due to concerns of hemodynamic instability and as such no re-entrant VT mapping data was acquired. These patients with 'pace-mapping only' were included to increase the sample size and allow to evaluate mapping accuracy in a cohort of more advanced myocardial disease. The study received a favourable opinion from the local research ethics committee (REC reference 14/LO/0360). All patients provided written informed consent.

### Electromechanical wave imaging

**Fig. 1:**
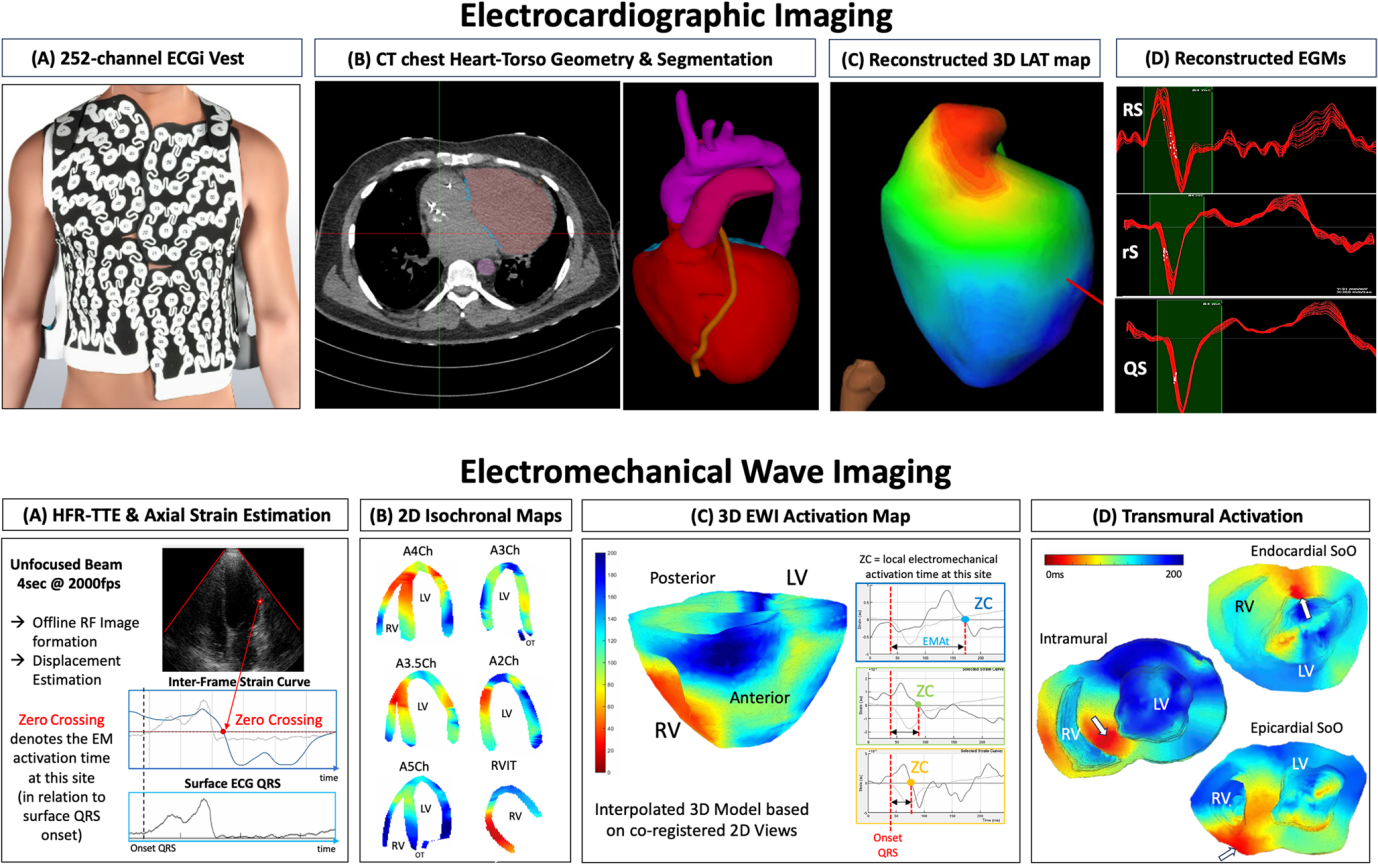
ECGI and EWI Mapping. Top: (A) ECGI was acquired using the CE marked CardioInsight System with disposable vest with 252-channels for body-surface potential recording (Panel A: reprinted with permission from Medtronic) and combined with (B) CT chest to allow segmentation of heart-torso geometry. (C) Temporal signal averaging, reconstruction of epicardial potentials and activation mapping was performed using the proprietary mapping software. (D) EGM morphology and slew rates were reviewed as surrogates for endo- vs. epicardial origin of the arrhythmia. Bottom: (A) High Frame Rate EWI 2D ultrasound data was acquired using a clinical transthoracic transducer. Acquisition, beam forming, ECG co-registration, electromechanical activation mapping and SoO definition followed a standardised semi-automated workflow (B) Site of earliest activation on any 2D view was defined as SoO (C) 2D isochronal maps were co-registered for 3D rendering (D) Examples of EWI mapping of SoO along the endo-epicardial axis showing an endo-, intramural and epicardial SoO, respectively

EWI utilizes the electro-mechanical coupling in the myocardium and the resulting minute electromechanical strain to infer to the electrical activation sequence, which precedes the electromechanical activation by a few milliseconds. Specifically, EWI uses incremental axial strain estimation at extremely high frame rates (100–200 times faster than the conventional echocardiography) to identify the first “zero crossing” (ZC) on the strain curve at each recording site. The ZC indicates the timepoint the myocardium switches from diastole (positive) to systole (negative strain) and is then related to the surface QRS, which serves as timing reference. In other words, the ZC is thought to represent the time when the myocardium at this site starts to “shorten” and thus can be used to define the onset of the local electromechanical activation as the corresponding surrogate for local activation time of conventional electrical activation maps. High spatio-temporal resolution EWI mapping is based on these high-frame-rate acquisitions and an accurate strain imaging technique (× 100 more accurate than available “speckle tracking” techniques in clinical echocardiography) capable of estimating the small regional interframe deformation to record precise electromechanical activation maps. One of its greatest advantages is that it can map the deformation transmurally, a unique mapping feature not afforded by any other clinical mapping tool. EWI data in this study was acquired transthoracically, although in theory it could also be used in similar fashion with trans-oesophageal (TOE) or intra-cardiac echocardiography (ICE), and analyzed as previously reported [5–7]. In brief, a standard phased-array transducer was used (64 element aperture, 2.5 MHz, 0.32 mm pitch, e.g. ATL P4-2, Philips, Andover, MA), connected to a non-commercial research ultrasound system (Vantage 256 of Verasonics Inc., Kirkland, WA, USA), and controlled by a custom-made Matlab script (MathWorks®) developed by Ultrasound Elasticity and Imaging Laboratory of the Department of Biomedical Engineering, Columbia University. Image acquisition for VA-mapping followed a standardized process as previously described for this indication [5]: Image acquisition involved an ultrasound sequence composed of 2 s B-mode (for anatomical imaging) followed by a 4-s high-frame-rate single diverging wave at 2000 Hz with a field of view of 128 scan lines spanning 90° (for activation mapping). Images were acquired with simultaneous ECG in at least 6 standard views in all patients (4-, 5-, 3.5-, 3-, 2-chamber and RVIT), shown in Fig. 1 Panel B (bottom row). Additional views were acquired if needed based on VE/VT ECG morphology (shown in Supplement Figure 1.1). Beam forming was performed offline and acquired B-mode and ECG were segmented to the area (ventricular myocardium) respectively beat (VE/VT or paced beat) of interest. Electromechanical wave mapping was then undertaken for each view separately with local electromechanical activation defined as time point of the downward zero crossing (ZC) on the incremental axial strain curve. For each view, approximately 150–300 points in the segmented mask were manually selected and reviewed, and the ZC annotated using a semi-automated detection algorithm. A Delaunay triangulation-based cubic interpolation was applied to the 2D scatter activation time values to achieve a continuous isochrone pattern throughout the entire myocardial mask. Subsequently, all views were co-registered based on surface ECG to align respectively synchronise to the onset of QRS (VE/VT) or pacing spike (paced beats) as a common timing reference. SoO in EWI maps was defined as the earliest activation in any view following co-registration of surface ECG VE/VT respectively paced beats and located on a biventricular 24-segment model to define “anatomical SoO” as well as along the transmural axis to define “axial SoO” (i.e. endo vs mid vs epicardial) (see Fig. 1). The segmental model and segment allocation per echocardiographic views are shown in Supplement Figure S1.

Within the imaging plane, this approach results in an axial resolution estimated at 0.0385 mm, lateral resolution at 0.43 mm at 5 cm, 0.84 mm at 10 cm, and 1.4 mm at 15 cm imaging depth and temporal resolution at 0.5ms (2000 fps) for EWI.

### Electrocardiographic imaging

ECGI was performed with the CardioInsight™ V3.5 3D mapping system. A single-use 252-electrode vest was applied, and torso-heart geometry was acquired with a non-contrast CT chest. CT segmentation of the vest, torso, and cardiac anatomy was performed using the proprietary segmentation software. Body surface potentials were recorded with a sampling rate of 1000 Hz. Epicardial unipolar electrograms (UEGMs) were reconstructed (low pass filter of 100Hz) and activation maps were generated using the maximum negative dVdt as LAT detection criteria. For all documented VE/VT morphologies as well as paced beats, temporal signal averaging was performed if feasible to improve signal-to-noise ratio using a correlation coefficient threshold of 0.93. The site of earliest activation on isochronal maps was defined as SoO. The employed ECGI approach reconstructs epicardial EGMs, as such only surrogates were available to estimate the SoO along the endo-epicardial axis: the morphology of the reconstructed UEGM at the SoO with QS morphologies was interpreted as supporting an epicardial SoO and rS as non-epicardial. Additionally, the slew rate of the UEGM (mV/ms) at the SoO was measured. All measurements were made in the CardioInsight™ software.

### Electro-anatomical mapping

The mapping and ablation procedure was performed as per routine clinical care and preference of the operator. A combination of conventional mapping features (site of earliest activation in bi- or omnipolar contact mapping supported by UEGM morphology) and/or complementary pace mapping and/or successful ablation site was defined as ground truth for anatomical SoO. While there was no uniform definition for a pace map cut-off indicative of SoO, a match of at least > 90% was required to be considered as acceptable correlation, as applied in previous studies [8, 9]. Diagnosis of intramural SoO was based on intramural coronary venous mapping or suspected based on combined RV-LV, respectively endo-epicardial activation mapping features. An epicardial origin was diagnosed by direct epicardial mapping (or epicardial pacing) or, in the absence of epicardial mapping, suspected if there was no pre-QRS activation on the endocardial maps and/or broad early areas with r wave in unipolar EGMs were recorded and/or no suppression with RF ablation was achieved.

For cases employing RV endo- or LV epicardial pace maps as surrogates of focal activation sources, anatomical cardiac CT models were reconstructed, and 3D lead position was extracted using threshold-based segmentation (Adas3D™, Galgo Medical). The lead position was marked on the 3D model and employed as surrogate for the focal SoO.

### Comparison of EWI vs ECGI activation times

Given the fundamentally different approach of EWI to traditional arrhythmia mapping modalities—electromechanical activation vs electrical propagation—we also aimed to quantify the difference in activation times (i.e. the electromechanical delay) between the two. To allow for a systematic comparison, the open-source code UNISYS for bullseye generation [10] was adapted to this ECGI dataset and further modified to allow extraction of epicardial activation times of the 3D EWI models. It was then applied to both epicardial EWI and ECGI activation maps to achieve a continuous standardized visualization of both biventricular models allowing for a direct comparison of EWI_AT_ and ECGI_AT_ on a global and segmental basis (see Supplement Fig. 2). Sub-analysis included comparison based on anatomical sites (RV vs LV, base vs midventricular vs apical segments) as well as between subgroups with and without structural heart disease.

### Statistical analysis

Continuous variables are expressed as mean ± standard deviation if normally distributed or median ± IQR if non-normally distributed and compared using Student’s *t*-test or Mann–Whitney *U* test as appropriate. For multiple comparisons, analysis of variance (ANOVA) was performed. Categorical variables are displayed as frequency counts and percentage. Statistical significance was set at α 0.05 and corrected for multiple tests if indicated. Statistical analysis was performed using IBM SPSS Statistics 29.

## Results

This was a single-centre cohort study including a total of 33 patients. Baseline demographics are reported in Table 1. Of these, in 24 patients, focal VE/VT activation mapping with EWI and ECGI with subsequent ablation was performed and non-invasive mapping was compared to contact mapping as gold standard. In 9 patients with scar-related re-entrant VT, but not in VT at the time of EWI/ECGI, pace maps using the intracardiac leads were acquired (including three patients with both LV and RV paced maps) and the site of lead position on CT was employed as reference for SoO. In one patient with VE-induced VF and ECGI-guided ablation, no VEs were present at the time of the EWI scan and no EWI-VE mapping was feasible. A total of 36 ECGI maps and 35 EWI maps were available for comparison (including paced maps) involving 12 (33.3%) endocardial, 11 (30.5%) mid-myocardial, and 13 (36.1%) epicardial sites of origin. Conversely, 11 (30.5%) localized to the RV, 17 (47.2%) to the LV (including the papillary muscle VE/VTs and LV paced maps), and 8 (22.2%) to the septum. Of the cases involving intramural sites of origin, six related to the septum and the remaining five to a left ventricular free wall or LV papillary muscle (individual results per subjects are reported in Supplement Table 1).

**Table 1:** Baseline demographics

	*N* = 33*
Gender	18 (54.5%) male
Age (years)	53 ± 17
BMI (kg/m^2^)	26 ± 4.6
Aetiology	
- DCM	16 (48.5%)
- ACM	4 (12.1%)
- HCM	2 (6.1%)
- MVD	3 (9.1%)
- ICM	2 (7.1%)
- Idiopathic	6 (18.2%)
LVEF	41 ± 17%
RVEF	53 ± 14%
LGE in CMR	20 (60.6%)
* - mid-myocardial*	6 (18.2%)
*- sub-epicardiac*	2 (6.1%)
- complex pattern**	9 (27.3%)
*- transmural*	2 (6.1%)
*- RV only*	1 (3%)
VE burden	27.2 ± 11.3%
Multifocal VE/VTs^#^	10 (30.3%)
Anti-arrhythmic drugs	
- BB or CCB^	28 (84.8%)
- Hydroquinidine	2 (6.1%)
- AAR IC	2 (6.1%)
- Amiodarone	7 (21.2%)
- Mexiletine	2 (6.1%)
Previous Ablation	13 (39.3%)

^*^Including all patients for VA and pace mapping, in three patients both RV and LV pace maps were acquired

^**^Different scar patterns across different segments

^#^Documented on 12-lead ECG or Holter

^Stand-alone or in combination with AARs

*DCM* dilated cardiomyopathy, *ACM* arrhythmogenic cardiomyopathy, *HCM* hypertrophic cardiomyopathy, *MVD* mitral valve disease, *ICM* ischemic cardiomyopathy, *LGE* late gadolinium enhancement, *VE/VT* ventricular ectopy/tachycardia, *BB* betablocker, *CCB* calcium channel blocker, *AAR IC* Class IC anti arrhythmic drugs (i.e. flecainide)

### EWI mapping results

In 80% (28/35 maps), the SoO was mapped to the correct anatomical segment. Failed anatomical localization occurred more often in right-sided SoO including patients with postero-septal RVOT VE/VT, free wall RV-VE, and RV paced maps in a dilated, aneurysmal RV in the context of advanced ARVC. Furthermore, in one patient with a papillary muscle VE, EWI mapped the origin to an intramural site in an adjacent segment to the exit site identified on the electro-anatomical map. In another case with dilated cardiomyopathy and epicardial LV-paced maps, diffuse early activation was detected over two segments on the LV lateral wall, but no clearly defined SoO was identified that would have allowed for more precise localization of the anatomical origin.

Axial SoO was correctly identified in 77.1% (27/35 maps). These “failed” cases included four maps with a diffuse early activation at the SoO without clear endo-epicardial differentiation. Failure was more common for endocardial SoO (including two cases of postero-septal RVOT) with only 54% of these correctly identified compared against 90.9% correctly located mid-myocardial and 84.6% epicardial SoOs (see Supplement Table 1 for detailed for individual results).

### ECGI mapping results

ECGI correctly identified the segmental SoO of the dominant VE/VT respectively pacing site in 77.8% (28/36) of the maps. ECGI failed in patients with septal VE/VT, which are not represented on the ECGI map, and located the dominant exit sites of papillary muscle VE/VTs to adjacent segments. In patients with papillary muscle arrhythmias and one patient with previous mitral valve repair, a second consistent, but less frequent morphology locating to the (left) ventricular outflow tract or the anterior/anteroseptal mitral annulus, respectively, was recorded by ECGI but not mapped intra-procedurally. In 4 patients with structural heart disease, ≥ 3 morphologies were captured by ECGI but only for the dominant VE/VT was a corresponding contact map acquired and targeted with ablation.

ECGI surrogates for endo-epicardial differentiation were assessed in all patients. There was no statistically significant difference between slew rate (*p* 0.534) for endo-, mid-, and epicardial SoO. QS was the most common UEGM morphology (in 77.8% of all maps). Numeric results are reported in Table 2.

**Table 2:** ECGI surrogates for endo-epicardial differentiation

SoO*	Slew Rate (mV/ms)	UEGM Morphology
Endocardial	0.32 ± 0.21	9/12 (75.0%) QS
Midmyocardial	0.37 ± 0.16	8/11 (72.7%) QS
Epicardial	0.29 ± 0.17	11/13 (84.6%) QS

^*^As defined by electro-anatomical contact mapping/ablation or pacing site

*UEGM* unipolar electrogram, *SoO* site of origin

### Comparison of localization accuracy of EWI vs ECGI

Comparing EWI vs ECGI maps, there was no statistically significant difference between the proportion of patients that were mapped to the correct anatomical segment using a modified AHA segment model (80% vs 77.8%, p ns) and employing contact mapping and pacing site as ground truth. However, only in two cases (5.5%), both modalities failed whereas for all others one modality localized correctly whereas the other did not. In 61.1% of cases, EWI and ECGI agreed correctly on the anatomical segment.

Of the two cases which yielded discrepant results for both non-invasive modalities, one involved a lateral papillary muscle arrhythmia in which both EWI and ECGI mapped the focus to an adjacent, but not identical, segment compared to the electroanatomical activation map. Subtle morphological variations in the ectopic beats suggested a shared origin with variable exit sites. The earliest activation on the contact maps was only −12 ms pre-QRS, indicating a deeper, possibly intramural source. Ablation at this site led to temporary suppression of the ectopy, but recurrence during follow-up further supported the hypothesis of a deeper focus within the basal portion of the papillary muscle. It is conceivable that EWI captured the intramural source, the contact map identified the endocardial exit site, and ECGI reflected the epicardial breakthrough. However, without direct intramural recordings, this remains speculative. The second case was a right ventricular pace map in a patient with severe RV dilation and aneurysmal remodelling due to advanced ARVC. The lead was positioned in the mid-to-high septum. ECGI does not cover septal segments and mapped the epicardial breakout site higher, towards the RVOT, while EWI mapped it more apically than the lead insertion site on CT. The discrepancy in EWI localization may have resulted from imaging plane misalignment within the markedly dilated and morphologically distorted RV. In such settings, 2D echocardiographic views may intersect the RV walls obliquely and/or misalign with the standard segmental model due to altered chamber orientation and geometry.

While EWI correctly derived the axial SoO in 77.1% of the cases, surrogates for endo-epicardial differentiation from ECGI did not show a significant difference between endo-, mid-, and epicardial sites of origin (see Table 2).

**Fig. 2:**
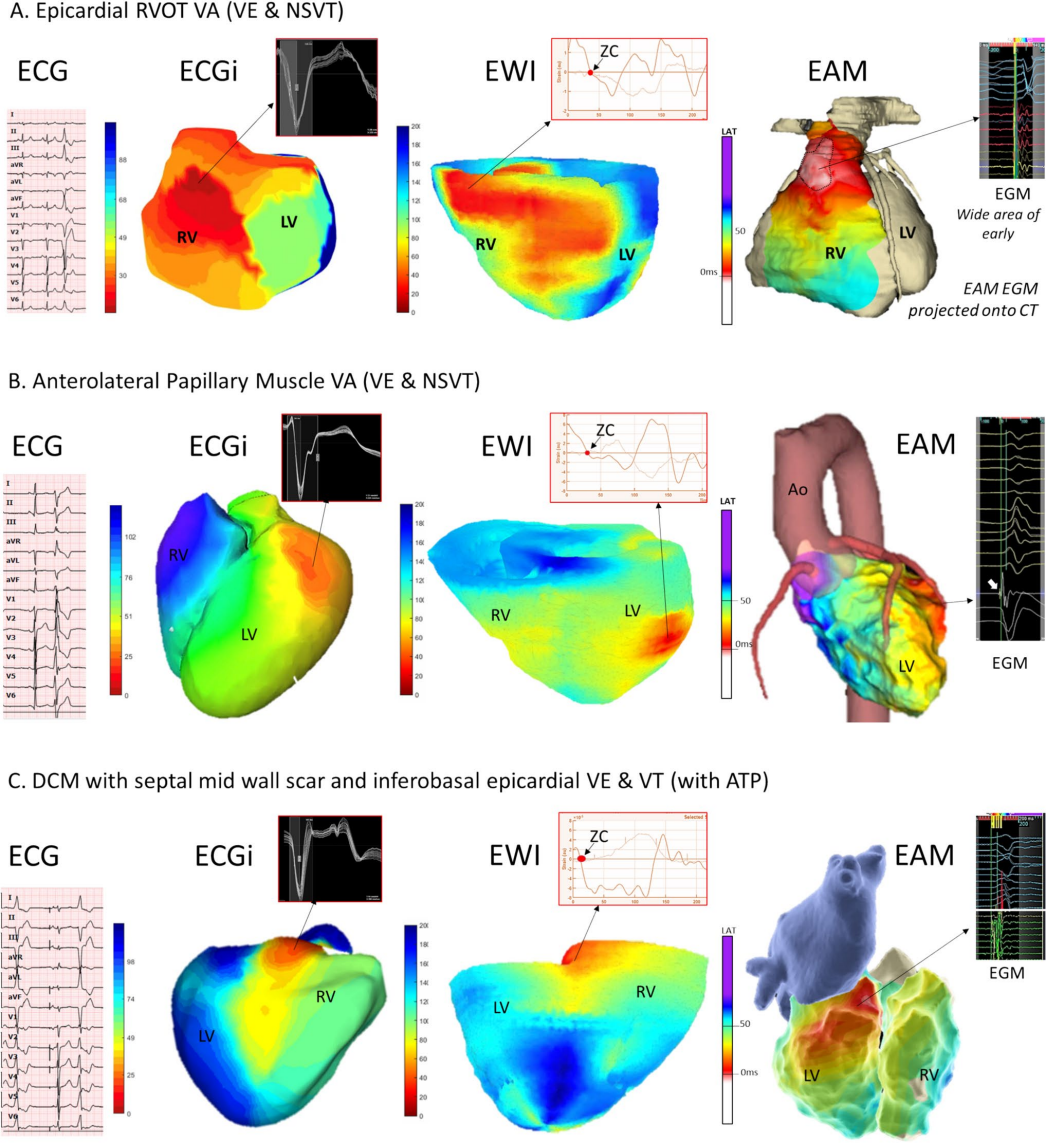
Case examples of side-by-side comparison of activation mapping of ventricular arrhythmias with ECGI, EWI and electro anatomical mapping (EAM) as reference and respective EGMs. Top: An epicardial anterior RVOT focus in a patient with suspected ARVC correctly identified in both non-invasive modalities. EWI correctly mapped it to the epicardium. Middle: An anterolateral papillary muscle VE/VTs with acute suppression with endocardial ablation but later recurrence. ECGI projects the epicardial break through site to the more basal segment compared to EWI. EWI mapped the earliest activation to an intramural SoO. Bottom: A DCM patient with septal and inferoseptal mid-wall scar. An epicardial focus was suspected based on only minimal prematurity on the endocardium with a relatively wide area of early. ECGI localized the earliest site right to crux cordis, as did the EWI although with a more pronounced preferential conduction towards the right ventricle compared to the contact map

**Fig. 3:**
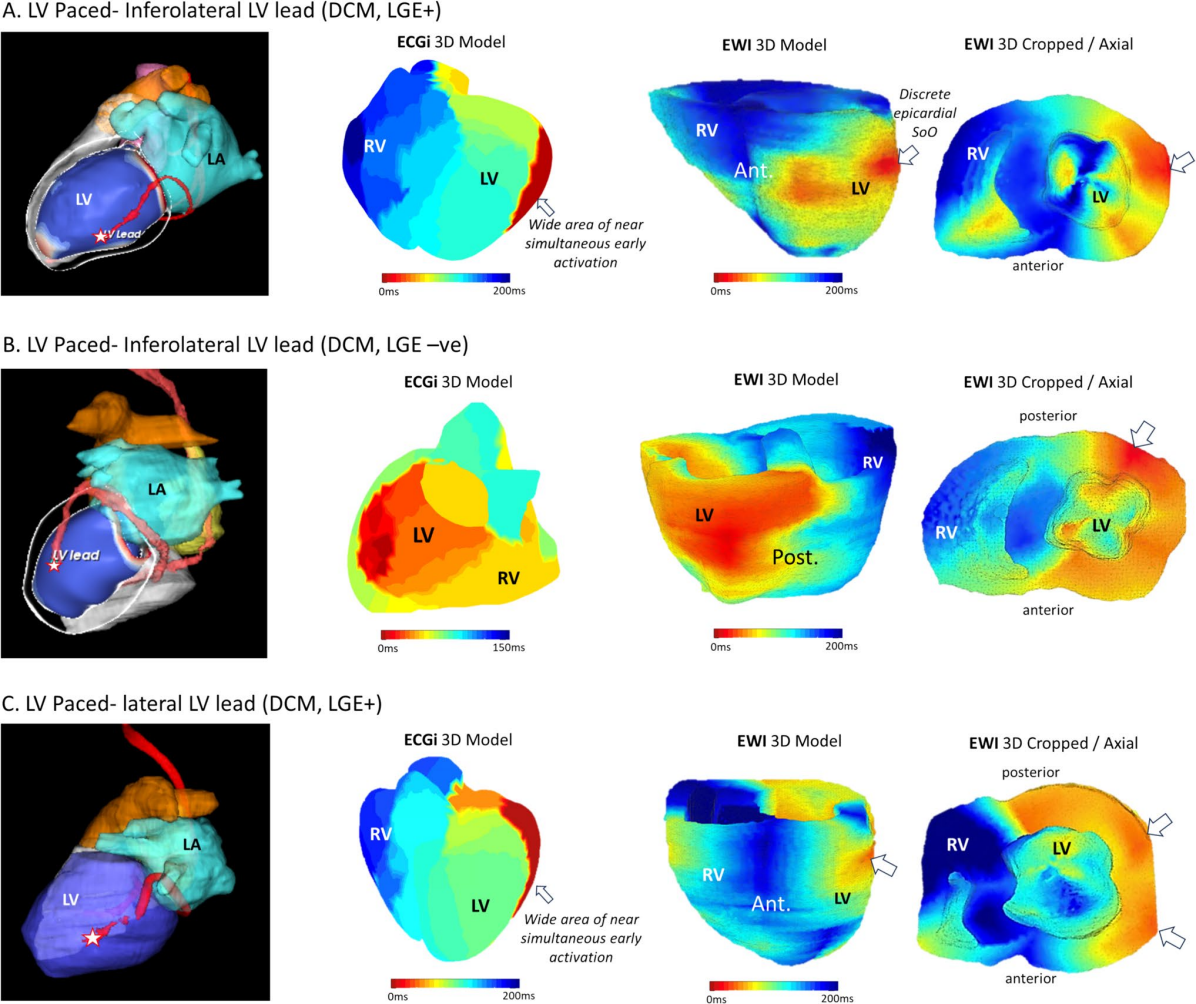
Case examples of epicardial LV Pacing sites for three patients with CRT. Left: 3D CT models with pacing lead position in red/pink on the inferolateral (**A**, **B**) and lateral (**C**) LV wall. Middle: ECGI 3D model (red denoting earliest activation)—cases **A** and **B** show wide areas of near simultaneously activating early sites encompassing the true SoO as well as adjacent segments with appearance of an artificial line of block towards the anterior segments. Case 2 is more discrete and correctly localized the SoO to the inferolateral mid ventricular segment. Right: Corresponding EWI 3D model. In cases **A** and **B**, EWI correctly identified the epicardial left ventricular pacing site with a much more discrete early activating area compared to ECGI. In case **C**, the left lateral wall appears diffusely early and the true SoO may not have been captured. A differential diagnosis would be a significant electromechanical latency between electrical and electromechanical activation

**Fig. 4:**
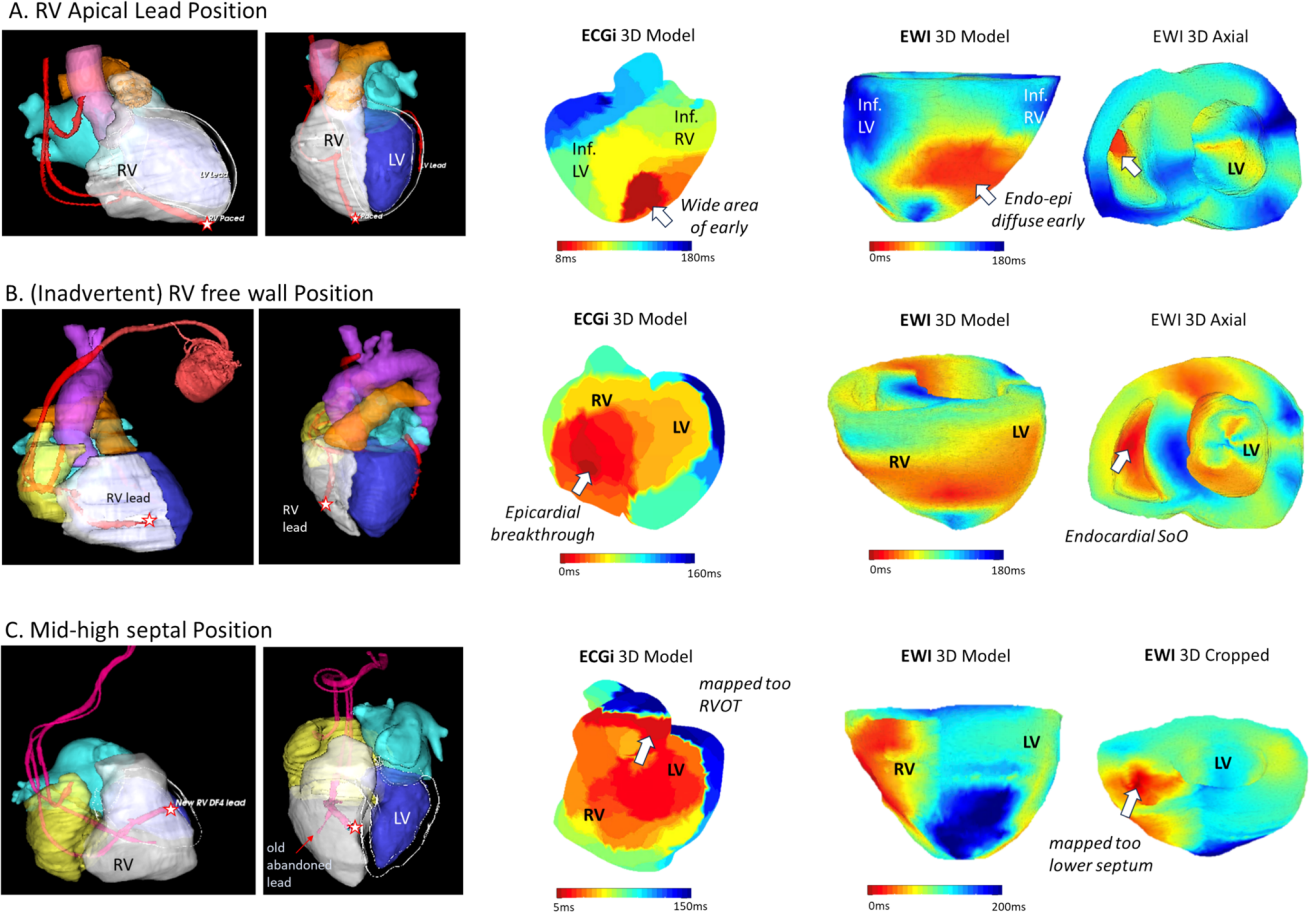
Case examples of endocardial RV paced models with three different RV lead positions. Left column: 3D CT models with leads in red/pink inserting in the RV apex (**A**. top), inadvertent placement on the anterior wall (**B**. middle) and mid-high anteroseptal position (**C**. bottom). Middle column: ECGI 3D (red denoting earliest activation) demonstrating correct localization in the apical and free wall position but projecting the epicardial breakthrough of the mid-septal pacing site too high to the RVOT. Right: corresponding EWI 3D models: (**A**) The map of the RV apical paced site shows a diffuse early activating area on both endo- and epicardium failing to clearly demonstrate the capture site to the endocardium. **B** The RV free wall pacing site appears earlier on the endo- than epicardium but projected slightly more lateral than the lead position on CT. **C** In the enlarged aneurysmatic RV in a patient with ARVC, the septal lead position is correctly identified and mapped to the RV endocardium but more apical compared to the CT lead position

Figure 2 shows three case examples of corresponding three-dimensional EWI, ECGI, and electro-anatomical maps of ventricular activation maps. Figures 3 and 4 illustrate three case examples of corresponding pace-maps of EWI, ECGI, and the CT model with RV endocardial and LV epicardial pacing lead position as reference for the impulse origin.

### Comparison of EWI vs ECGI activation times

Comparison of the epicardial activation times of ECGI maps (ECGI_AT_) versus corresponding EWI maps (EWI_AT_) revealed a mean difference of 45 ± 28ms across all segments (with ECGI_AT_ preceding EWI_AT_ in > 90% of segments). Sub-analysis of global ECGI_AT_ and EWI_AT_ according to the presence of scar and LVEF revealed a significant difference (*p* < 0.001) between subjects with scar (ECGI_AT_-EWI_AT_ difference 50 ± 29ms) and those with impaired LVEF but no scar (ECGI_AT_-EWI_AT_ difference 39 ± 25ms) as well as structurally normal hearts (ECGI_AT_-EWI_AT_-difference 33 ± 21ms). Post hoc pairwise comparison was significant for LGE + ve subjects vs. structurally normal hearts ( *p* < 0.001) and LGE + ve versus impaired LVEF without scar ( *p* < 0.001) but not significant for impaired LVEF without scar vs structurally normal hearts (39 ± 25 vs 33 ± 21ms, *p* 0.111).

Further sub-analysis comparing basal, mid, and apical segments showed largest activation time differences in the apical segments with a mean difference of 51 ± 28ms including all patients. Tabulated results for all comparisons and subgroups are shown in Table 3.

**Table 3:** Global and segmental differences in ECGI vs EWI activation times

	All		LGE + ve	LVSD & LGE-ve	Structurally normal	p-value**
	*N* = 33		*N* = 20	*N* = 4	*N* = 9	
Global	45 ± 28ms		50 ± 29ms	39 ± 25ms	33 ± 21ms	< 0.001
Basal-Seg	38 ± 27ms	*p* < 0.001*	41 ± 27ms	43 ± 30ms	27 ± 22ms	< 0.001
Mid-Seg	45 ± 27ms		51 ± 28ms	36 ± 27ms	35 ± 22ms	< 0.001
Apical-Seg	51 ± 28ms		59 ± 29ms	39 ± 18ms	36 ± 19ms	< 0.001
RV-Seg	46 ± 28ms	*p* ns*	51 ± 29ms	44 ± 28ms	34 ± 22ms	< 0.001
LV-Seg	43 ± 28ms		50 ± 30ms	35 ± 23ms	31 ± 21ms	< 0.001

All values reported in ms, values per category represent mean ± SD difference in AT between ECGI and EWI maps

^*^p value for comparison basal vs mid vs apical segments r, respectively, RV vs LV

^**^p value for comparison of basal, mid, apical, RV and LV segments across the three groups of ‘LGE+ve’, ‘LV systolic dysfunction (LVSD) & LGE-ve’ and ‘structurally normal hearts’ within the respective category

*ns* non-significant, *LGE* late gadolinium enhancement, +ve positive, -ve negative, *LVSD* left ventricular systolic dysfunction, *Seg* segment

## Discussion

This is the first study to compare two advanced, but fundamentally different, approaches to non-invasive clinical global activation mapping of VAs in a mixed cohort of scar-related and idiopathic focal ventricular arrhythmias and pace maps. The main results of this study are as follows:

- Both EWI and ECGI identified the correct site of origin in the majority of cases and each modality was found to have distinct site-specific benefits but also shortcomings.
- EWI allows direct, tissue-based mapping which provides good results for differentiating endo from epicardial site of origin and potentially could be integrated in routine echocardiographic exams without requiring additional hardware or additional specialized imaging.
- EWI in its current form is challenging for mapping of rare and/or multifocal arrhythmias and 2D acquisition leaves interplanar gaps that may harbour the true site of origin. Post-processing is time consuming and still requires substantial manual input.
- ECGI offers high-resolution panoramic real-time mapping that allows mapping of even rare isolated VEs and facilitates multifocal arrhythmia mapping with extensive literature supporting its use for this purpose. It can be employed in real-time, and is thus suitable for intraprocedural guidance.
- ECGI mapping, using epicardial reconstructed unipolar EGMs, is of limited value in septal or intracavitary structures (for example, papillary muscle) and does not afford reliable distinction between endo- and epicardial SoOs. It requires special equipment and advanced cardiac imaging (CT or CMR), adding cost and/or exposing the patient to radiation.

### Non-invasive ventricular activation mapping: EWI and ECGI as complementary options

In our study, we found clear site and modality specific advantages and disadvantages both for ECGI and EWI (summarized in Supplement Table 1) which has important implications for their clinical use and implementation.

*Electrocardiographic imaging* is currently the only clinically established non-invasive 3D cardiac mapping technology, and several commercial systems have been approved for this purpose (for example CardioInsight™, Medtronic, USA; VIVO™, Catheter Precision, USA; Amycard 01C™, EP Solutions SA, Switzerland Acorys®, Corify Care ^(c)^, Spain). Comparative studies have shown reasonable agreement regardless of the cardiac source model employed [11]. Yet, the lack of reliable distinction between endo- and epicardial origins of the arrhythmia is generally considered a short-coming and our study employing epicardial reconstructed EGMs was in agreement with this. Estimated surrogate markers did not reliably predict the axial SoO. Another acknowledged limitation of ECGI is the absent representation of septal segments and intracavitary structures such as papillary muscles. Inclusion of patients with arrhythmias originating from these sites contributed to the overall lower success rate of ECGI in our cohort when compared with other reports [4, 12]. The ECGI approach employed here could only identify the epicardial breakthrough of these septal or intracavitary arrhythmias, which may locate to an adjacent segment and not represent the true SoO. Such failed localization could have negative consequences if a non-invasive ablation technology is employed to target these sites. Similar concerns have been raised for the use of ECGI for ventricular substrate mapping [13, 14].

This is contrasted by the superiority of ECGI in terms of global, real-time single-beat activation mapping, which allows even in rare but highly proarrhythmogenic events, i.e. VE-induced ventricular fibrillation, to define the area of interest and can be used to guide intraprocedural mapping—arguably one of its most important applications in clinical practice. Secondly, ECGI is a useful tool if multi-focal VEs are present which are a challenge for any sequential mapping approach including invasive contact mapping. The option to identify multiple sites of interest and narrow down the area that requires detailed contact mapping can be vital for procedural success. Previous research reported that the use of ECGI may allow to significantly reduce mapping time and therefore procedure time [15, 16].

ECGI is an area of ongoing research. Advanced detection methods like the Bayesian technique were recently found to improve localization accuracy of ventricular ectopics [17]. Also robustness of imageless ECGI has recently been reported [18] and may facilitate integration in clinical workflows and reduce costs.

#### Electromechanical wave imaging

EWI is an echocardiography-based mapping modality that has been investigated both for arrhythmia mapping and optimizing cardiac resynchronization therapy [5, 19–21]. Its main advantage is that it affords true transmural mapping to differentiate endo-, mid-, and epicardial SoOs. This makes it a highly promising complementary diagnostic tool filling an important gap in current mapping technology which remains largely restricted to surface recordings. Also, in our study, it correctly identified the majority of deep septal and papillary muscle arrhythmias where ECGI failed. Conversely, one previous study by the Konofagou lab also showed that delayed activation of the papillary muscle in sinus may serve as a biomarker for arrhythmogenic valve conditions [22]. Yet, while the advantages are evident, limitations and challenges remain that need to be addressed prior to a more widespread use in clinical practice. The restriction to 2D-echocardiography risks “missing” the true SoO if it is located between the scanning planes as evident in four of the included patients in our cohort. In these patients only a diffuse area of early activation was identified rather than a well-defined focal site that would allow precise localization and distinction of endo- or epicardial origin. Also, accurate visualization of the right ventricular segments, in particular if significantly dilated, as well as the outflow tract is challenging with transthoracic echocardiography, and in our cohort, failed SoO localization was more common in right ventricular foci. This difficulty is compounded by the complex, crescentic geometry of the RV, which wraps around the LV, making consistent and precise co-localization between the 2D imaging plane and a standard anatomical model challenging, especially when atypical or modified views are required. Precision and localization accuracy of this technology could be substantially improved by expanding it to true 3D full volume acquisitions. General feasibility of 3D EWI has been established during pacing and VT in a canine model [23] and healthy volunteers [24] but awaits application to clinical real-time arrhythmia mapping.

Another inherent challenge of EWI, independent of 2D or 3D acquisition mode, is that electromechanical waves may be distorted and dissociated at sites of scar. Inclusion of patients with more extensive scarring may also have contributed to the lower overall accuracy of EWI mapping in this cohort compared to the previously reported case series [5]. Additionally, throughout the cardiac cycle, numerous other mechanical phenomena may occur and cause “noise” on the strain curve interfering with tracing the electromechanical wave from the source. The ambiguity of strain curves which may have more than one zero crossing or none at all is an important challenge for fully automated EWI mapping. Currently, mapping still requires substantial manual input. Machine learning projects have been reported [25] and are in progress to address this.

#### Electrical and electromechanical activation times

The relationship between electrical and electromechanical activation times is a key consideration for the interpretation of EWI maps and an important distinction when comparing EWI to electrical mapping modalities such as ECGI. EWI activation times would be expected to occur later than ECGI activation times due to the inherent electromechanical delay (the time between local electrical depolarization and the onset of a measurable mechanical contraction). However, importantly, this delay is not uniform and is influence by multiple physiological but also technical factors. EMD differences can be observed between apex and base [26] and endo- and epicardium [27] even in normal hearts and may also vary depending on heart rate [28] and rhythm [29]. In structural heart disease, electromechanical coupling can be further disrupted by myocardial scarring and fibrosis and also modulated by altered loading conditions and heart failure [30]. The findings presented herein confirmed that ECGI-AT preceded EWI-AT in over 90% of segments with an average delay on a global level of 45 ± 28 ms. On a segmental level, EMD was significantly prolonged in segments with myocardial scar, to a lesser degree in those with LV dysfunction but no scar, supporting the hypothesis that electromechanical coupling is altered in diseased myocardium. While absolute EWI-AT values may be on average later than ECGI-AT, the relative earliest site identified on EWI maps can still accurately reflect the site of origin, and in the case of intramural sources may even precede surface electrical measurements.

The findings underscore the need to interpret EWI and ECGI as complementary rather than interchangeable modalities. Moreover, they also suggest that EWI may have additional value in characterizing electromechanical delay and dissociation in structural heart disease, especially as it can be applied in conjunction with a simple transthoracic echocardiogram. If validated in larger cohorts, EWI-ECGI-derived electromechanical delays may serve as a novel biomarker, with possible implications for early identification of patients at risk for PVC-induced cardiomyopathy or adverse remodelling. Future studies, including an ongoing project in our group using EWI and ECGI in baseline rhythms (SR or RVP), aim to more precisely quantify regional electro-mechanical delay and its relationship to scar and functional impairment.

However, several technical limitations must be acknowledged. Differences in spatial resolution, anatomical sampling, and depth of signal acquisition between EWI and ECGI introduce further complexity to EMD results. EWI can detect intramural electromechanical activation, whereas ECGI is confined to the epicardial surface. As such, EWI may detect early activation at depth not visible to ECGI leading to instances where EWI may appear earlier than ECGI despite the expected electromechanical delay. Conversely, EWI’s current restriction to 2D imaging planes requires interpolation and may artificially increase or decrease the delay at certain interpolated regions, yet this could be addressed in future studies as recent reports by the Konofagou group demonstrated successful single-beat 3D EWI [24]. In turn, ECGI, while offering global epicardial coverage of unipolar EGMs, lacks the spatial resolution to detect small pre-potentials or intramural sources and may therefore miss or mis-localized very early activation sites. These modality-specific characteristics likely affect the estimated EMD and justify the choice of a segmental, rather than point by point, comparison, at the cost of local resolution for EMD measurements.

Finally, we observed that activation time differences of ECGI_AT_ and EWI_AT_ were largest in apical segments. While this may reflect true physiological variation, it is also likely confounded by the complex motion of the apex, which presents known challenges for accurate EWI tracking using axial strain curves. This highlights the need for caution when interpretating EWI maps in apical regions using axial strain mapping.

### Limitations

This was a limited sample size of a mixed cohort of idiopathic and scar-related ventricular arrhythmias. The study was underpowered to demonstrate non-inferiority of EWI compared to ECGI for mapping of ventricular arrhythmias. Also, comparisons were performed based on a semi-quantitative segmental approach due to the inherent differences of the modalities challenging accurate co-registration as well as additional limitations introduced by transmural activation/intramural SoOs not amenable for a point-by-point comparison.

The three modalities involve separate acquisition systems with distinct reconstruction methods and do not share a spatial framework that would allow direct overlay or measurement between them without introducing co-registration errors. Also, contact maps and ECGI represent electrical activation on cardiac surfaces; EWI by contrast measured activation transmurally from endo- to epicardium within the 2D imaging plane, which inherently lacks a direct spatial correlate of intramural data in EAM/ECGI-derived surfaces to measure anatomical distances. In turn, ECGI models lack the septal segments making it impossible to measure distances for arrhythmias arising from these regions. Lastly, EWI lacks the anatomical landmarks traditionally employed for model alignment (aortic arch, pulmonary artery, pulmonary veins). EWI is also not respiratory gated. In turn, ECGI CT is acquired in inspiratory breath hold leading to further variability in geometry. Co-registration processes can therefore introduce additional spatial inaccuracies, which may misrepresent actual location differences and make any distance measure inherently uncertain.

The logistics and set-up of this study for non-invasive data acquisition did not allow for induction and mapping of re-entrant VTs due to concerns of hemodynamic instability. VT induction protocols were restricted to the electrophysiological laboratory with appropriate monitoring and resuscitation equipment available. As such, the results of this study for mapping of ventricular arrhythmias are limited to those with focal mechanism. Current prospective research projects are ongoing for EWI to address this shortcoming and undertake simultaneous EWI/EAM mapping directly in the EP catheter lab.

To assess the absolute difference in activation times, epicardial EWI and ECGI were compared to each other but not to contact mapping. Contact mapping data was frequently limited to one chamber only, mostly endocardial and point density remote from the area of interest often low, thus making it unsuitable for a direct comparison.

In this study, we employed an ECGI approach reconstructing epicardial unipolar EGMs and we only evaluated indirect features for endo-epicardial differentiation. The results cannot be extrapolated to alternative ECGI systems based on other cardiac source models that may offer combined endo-epicardial mapping.

Lastly, intracardiac mapping was performed using state-of-the-art equipment. Yet, the intramural space remains inaccessible with contact mapping and venous mapping is restricted by vascular anatomy. Thus, definition of the precise intramural site of origin remains challenging.

## Conclusion

Both EWI and ECGI correctly identified the SoO on a modified AHA model in the majority of cases with distinct site-specific advantages and shortcomings for each modality. EWI provided crucial information in determining the transmural origin which could not be localized by ECGI. ECGI had the advantage of affording mapping of multifocal and rare VA. EWI and ECGI could complement each other, and future research should evaluate if merging these technologies could yield superior results by combining the advantages of both and overcoming their respective limitations.

## Supplementary Information

Below is the link to the electronic supplementary material.

Supplementary file1 (DOCX 4267 KB)

Supplementary file2 (DOCX 35 KB)

## Data Availability

Due to contractual restrictions by Medtronic, no CardioInsight™ ECGI data can be shared.
