# Supplementary material for: Electromechanical wave imaging vs electrocardiographic imaging: a direct comparison of non-invasive ventricular activation mapping modalities
Source: J Interv Card Electrophysiol. 2025 Nov 24;69(3):467–79. doi: 10.1007/s10840-025-02156-y (PMC13009115; doi:10.1007/s10840-025-02156-y)
Supplement: Supplementary file 1 — Supplementary file1 (DOCX 4267 KB) [file 10840_2025_2156_MOESM1_ESM.docx]

**Supplement Figure 1 .**

**Standardized 24-Segment Models for Echo and ECGi for definition of SoO**

**1.1.Segmentation of echocardiographic views**


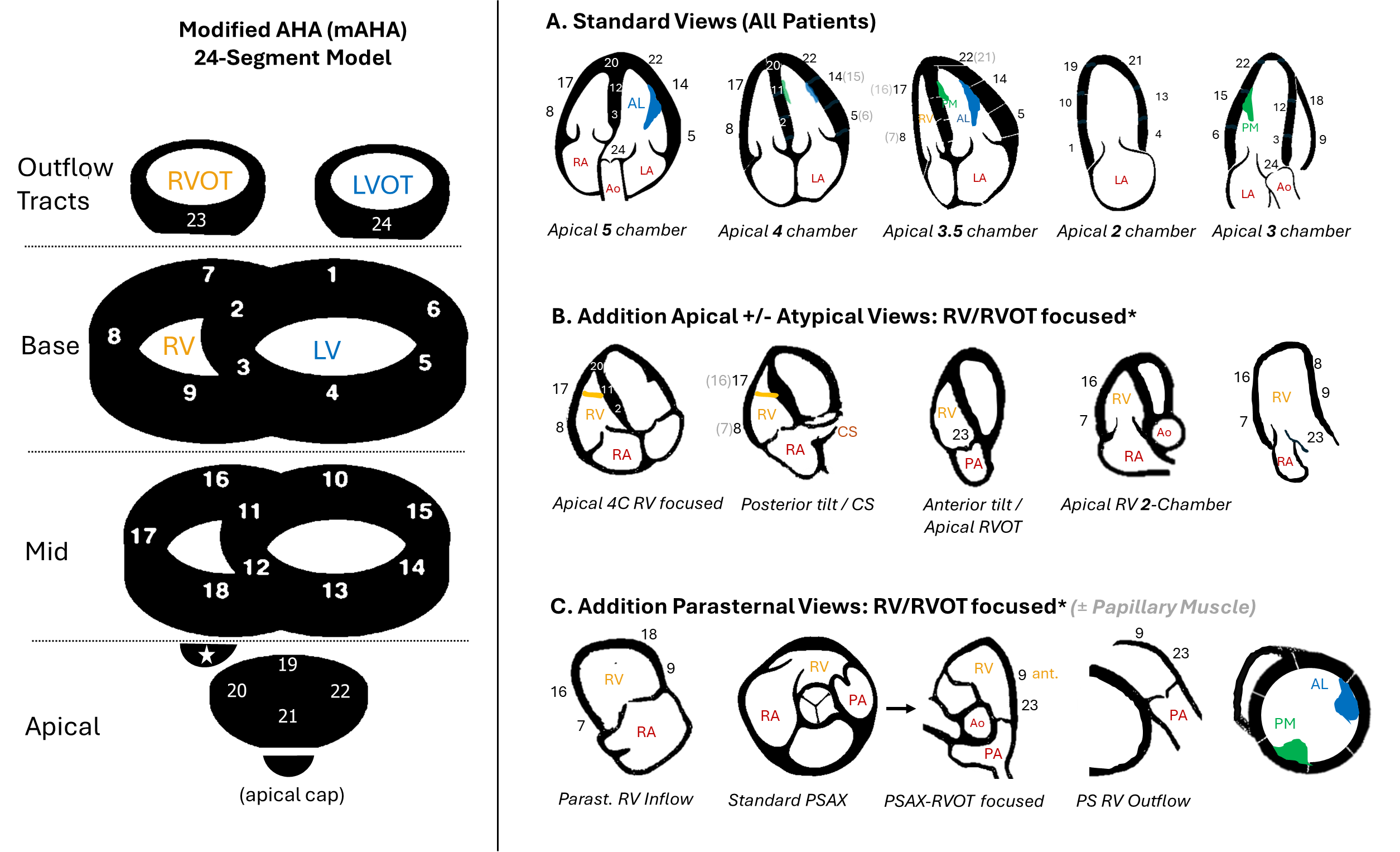


*The RV apical segment counted towards apico-septal segment (20) on the apical level. (*Supplement Fig 1.1. adapted from and reprinted from Tonko J et al.* *doi: 10.1016/j.jacep.2024.11.019*)

**1.2. Segmentation of CT-ECGI maps**


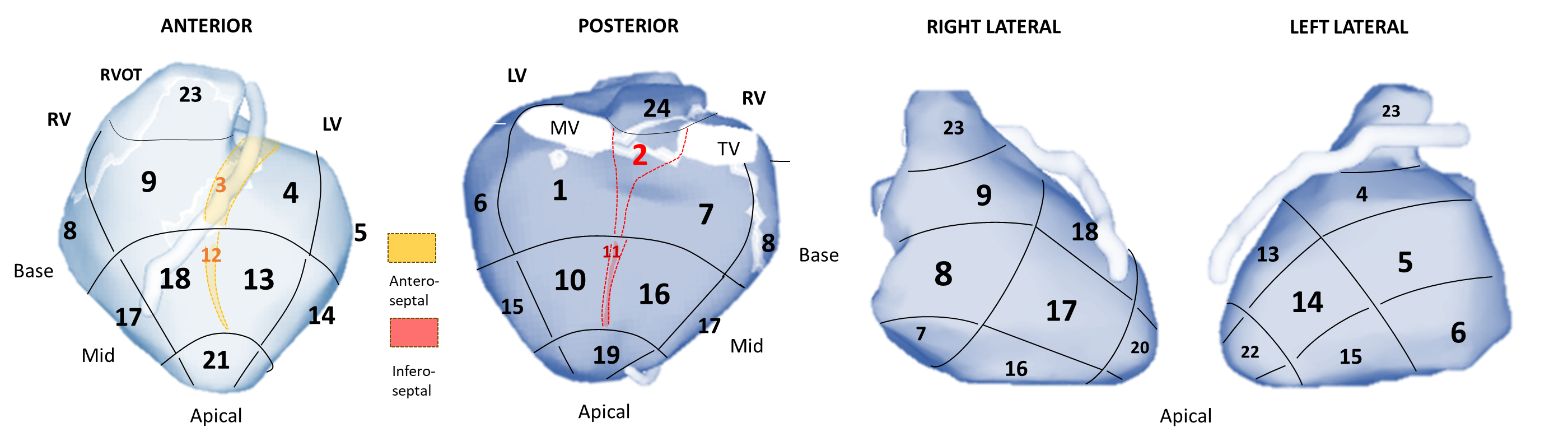


**Supplement Figure 2.**

**Bullseye Segmentation for Epicardial EWI and ECGI Activation Times**

Bullseye represents epicardial activation for ECGI and EWI. Mid and endocardial activation recorded by EWI are not represented

**
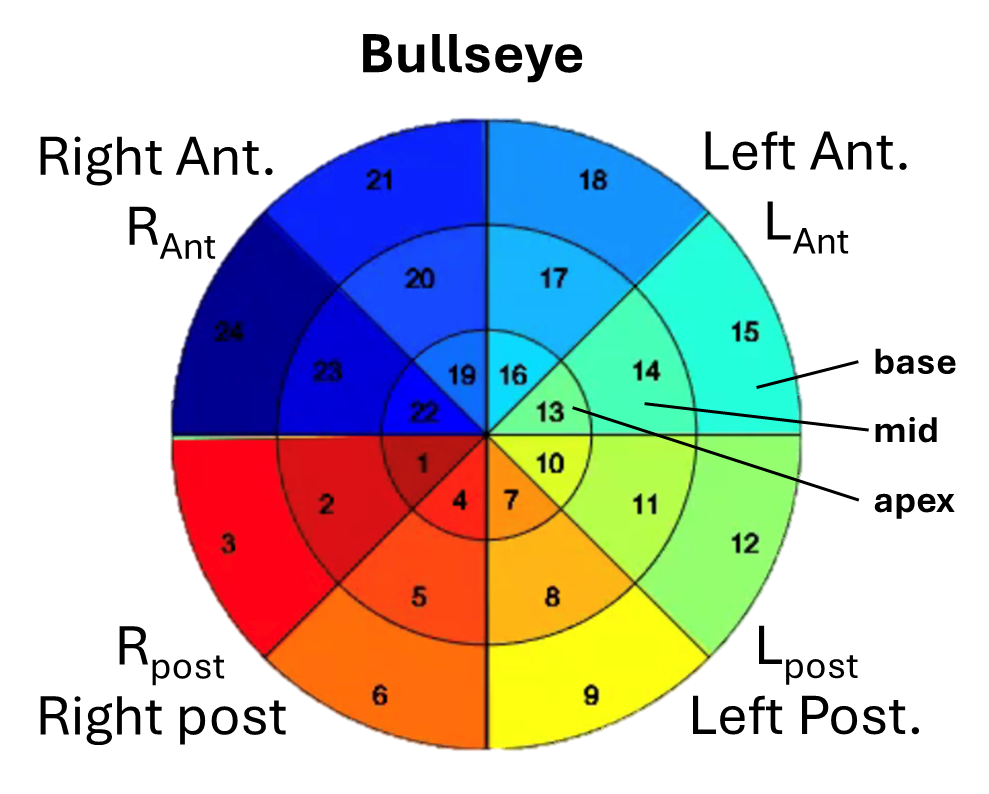
**

**ECGI**

**
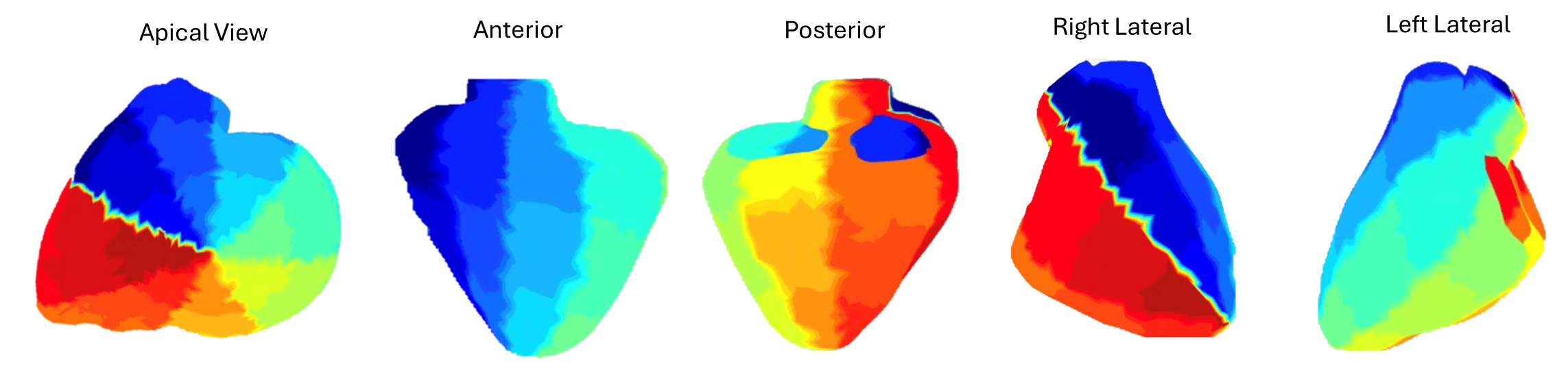
**

**EWI** **
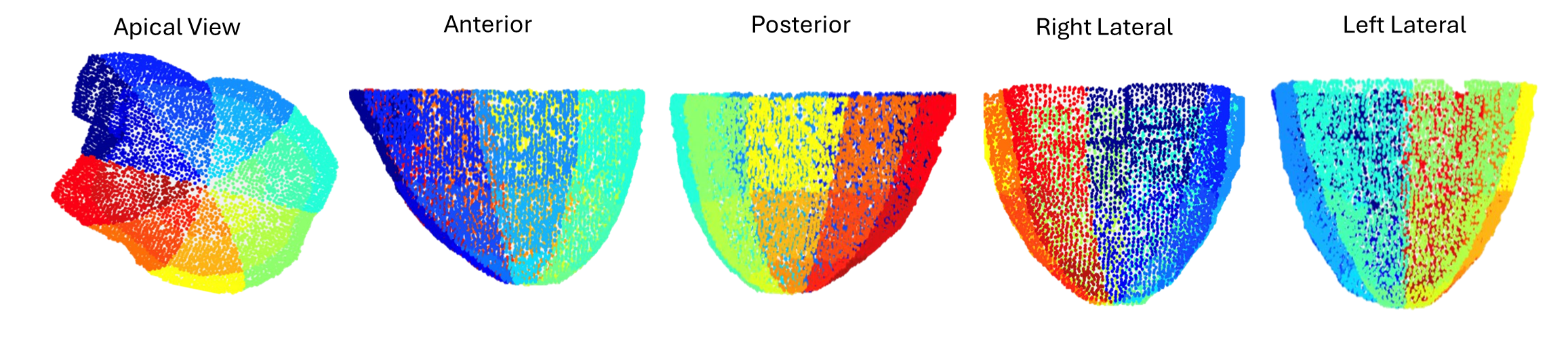
**
