# Supplementary material for: Electromechanical wave imaging vs electrocardiographic imaging: a direct comparison of non-invasive ventricular activation mapping modalities
Source: J Interv Card Electrophysiol. 2025 Nov 24;69(3):467–79. doi: 10.1007/s10840-025-02156-y (PMC13009115; doi:10.1007/s10840-025-02156-y)
Supplement: Supplementary file 2 — Supplementary file2 (DOCX 35 KB) [file 10840_2025_2156_MOESM2_ESM.docx]

**Supplement Table 1. Subject-level Summary of Origin of Ventricular Arrhythmias**

**PART 1.1. FOCAL VENTRICULAR ARRHYTHMIAS**

| Nr. | Aetiology | EAM Approach | SOO  EAM | EWI Corr* | ECGI Corr* | Acute Succ^ | % burden PRE | % burden POST | FU^§^  (Mo) | Comment |
| --- | --- | --- | --- | --- | --- | --- | --- | --- | --- | --- |
| 1 | DCM  Gene -ve | Endo-Epi | Epi LV free wall | Y/Y | Y | Y | 14%  ATP +shock (VT) | <0.1% | 23 | No further VT / ICD Tx |
| 2 | DCM (inflamm.) | Endo | Epi LV inferior / inf.septal  *(susp)* | Y/Y | Y | Y | 30%  + NSVTs | 10% | 6 | Medical Tx |
| 3 | DCM  (TTN) | Endo | Epi LV inferior base *(susp.)* | Y/Y | Y | P | 19%  + NSVTs | 4.5% | 19 | Ablated from endo only, uptitrate HF medication post-procedure |
| 4 | DCM (bystander CAD) | Endo/CS | Epi LV (summit) | Y/Y | Y | P | 50 %  + VF arrest | - | 11 | Only device FU –isolated VE, no VT |
| 5 | MVP | Endo | PPM  (LV) | Y/Y | N | Y | 38% | 1% | 3 |  |
| 6 | Idiop. VF | Endo-Epi | ALPM  (LV) | Y/Y | N | Y | 35%  & ICD shock (VF) | 12% | 15 | Medical Tx  (patient pref.) |
| 7 | DCM (?VE induced) | Endo | ALPM (LV) | Y/Y | N | P | 31%  + NSVTs | 30.7% | 6 | Underwent redo 15mo after first procedure – FU Holter pending (ECGs: no further VEs) |
| 8 | DCM  Gene -ve | Endo-Epi | IM LV (left lateral) | Y/Y | Y | Y* | 73% & Incessant MMVT | 34% | 16 | Medical Tx (pat. preference) |
| 9 | MVP | Endo | ALPV  (LV) | Y/N | N | Y | 34%  & ICD shock (VF) | 3% | 13 |  |
| 10 | Idiop. VE | Endo | LVOT | Y/Y | Y | Y | 37% | <0.1% | 20 |  |
| 11 | MVR | Endo | Endo LV (inf.- septal) | Y/Y | N | Y | 10% | 9% | 3 | Awaiting redo (patient preference) |
| 12 | DCM  Gene -ve | Endo | Endo LV (septal base) | N/Y | N | Y | 31% | 4% | 6 |  |
| 13 | DCM  Gene-ve | Endo | IM infero-septal (*Crux)* | Y/N | Y | N | 23.4%  + NSVTs, ATP (VT) | 8.6% | 2 | Medical Tx |
| 14 | DCM (RBM20, DSG2 VUS) | Endo-Epi | IM infero-sepal (*Crux)* | Y/Y | Y | P | 24% | 17% | 14 | Medical Tx |
| 15 | DCM  Gene -ve | Endo/CS | IM (septal) | Y/Y | Y | Y | 15% | 10% | 12 |  |
| 16 | DCM (?VE induced) | Endo/CS | IM (septal) | Y/Y | Y | Y | 22% | *n/a* | *n/a* | *No FU* |
| 17 | Idiop. VE | Endo/CS | IM (septal) | Y/Y | Y | Y | 33% | 27% | 8 | Awaiting redo |
| 18 | HCM | Endo/CS | IM (septal) | N/Y | Y | P | 16.3% | 8% | 7 | Asymptomatic, conservative Tx |
| 19 | ARVC | Endo-Epi | Epi RV | Y/Y | Y | Y | 33% | <0.1% | 25 |  |
| 20 | Susp. ARVC | Endo | Epi RV *(Susp.)* | Y/Y | Y | Y | 40% | 16% | 28 | Underwent redo – FU Holter 5 months after re-do: VE<0.1% |
| 21 | Idiop. VE | Endo | Endo RV (free wall) | Y/N | Y | Y | 27% | <0.1% | 10 |  |
| 22 | DCM (?VE induced) | Endo | RVOT | N/N | Y | Y | 29% | <0.1% | 7 |  |
| 23 | Idiop. VE | Endo | RVOT | N/N | Y | Y | 24% | <0.1% | 22 |  |
| 24 | Idiop. VF | Endo | Endo RV (MB) | n.a. | Y | Y | 13%  & shock (VF) | <0.1% | 19 | No further shocks |
| Abbr. ALPM Anterolateral Papillary Muscle, ARVC Arrhythmogenic right ventricular cardiomyopathy, CAD Coronary Artery Disease, CS coronary sinus, DCM Dilated Cardiomyopathy, Epi= epicardial, Endo = endocardial, FU = follow up, HCM hypertrophic cardiomyopathy, IM= intramural, MVP Mitral valve prolapse, MVR=Mitral valve repair, PPM posterior papillary muscle, RVOT=right ventricular outflow tract, TTN= Titin mutation, VE ventricular ectopy, VF ventricular fibrillation, VUS= Variant of unknown significance  *EWI Corr: “Y” = EWI in agreement with EAM (=correct), “N” = EWI not in agreement with EAM (=false) 🡪first position refers to transmural SoO (endo/mid/epi), second position refers to anatomical/segmental SoO. Example “N/Y” = failed detection along the endo-epicardial axis but correct anatomical segment  *ECGI Corr: “Y” = anatomical/segmental SoO in ECGI in agreement with EAM (=correct), “N” = not in agreement (=false)  ^Acute Success: Y = abolition of clinical VE/VT, P = partial if suppression but still residual isolated VEs (spontaneous or with Isoprenaline)  § FU: Follow up in months estimated as time between procedure and most recent Holter ECG / Device check | | | | | | | | | | |

**PART 2. SCAR-RELATED VT PATIENTS (RV and LV pacing via Implantable Device only)**

| Nr. | Aetiology | Scar Location | Pacing Site | EWI Corr* | ECGICorr* |  |
| --- | --- | --- | --- | --- | --- | --- |
| 25 | DCM (Chemo) | Inferior/  Inf.septal LGE | RVP Endo | Y/Y | Y |  |
|  |  |  | LVP Epi | N/Y | Y |  |
| 26 | ARVC | RV | RVP Endo | Y/N | N |  |
| 27 | DCM (LMNA) | Inferior/  Inf.lateral LGE | RVP Endo | Y/Y | Y |  |
|  |  |  | LVP Epi | Y/Y | Y |  |
| 28 | ACM ?Myocarditis | LV Inferior / septal + RV | LVP Epi | Y/Y | N |  |
| 29 | Burned-out HCM | Extensive patchy LGE in LV | LVP Epi | Y/Y | Y |  |
| 30 | DCM  Gene -ve | Mid-wall septal, inferior LGE | RVP Endo | N/Y | Y |  |
|  |  |  | LVP Epi | N/N | Y |  |
| 31 | ICM | Anterior MI | LVP Epi | Y/Y | Y |  |
| 32 | ICM | Inferior/ Inf.lateral MI | RVP Endo | N/Y | Y |  |
| 33 | DCM  Gene -ve | Mid-septal LGE | LVP Epi | Y/Y | Y |  |
| Abbr. ARVC Arrhythmogenic right ventricular cardiomyopathy, DCM Dilated Cardiomyopathy, HCM Hypertrophic Cardiomyopathy, LGE Late gadolinium enhancement, LMNA Lamin mutation, ICM Ischemic Cardiomyopathy, MW mid wall, MI Myocardial Infarction | | | | | | |

**Supplement Table 2. Advantages and Disadvantages of EWI and ECGI**

|  | | EWI | ECGI | |  |
| --- | --- | --- | --- | --- | --- |
| Advantages | - Cost-efficient bedside investigation - Low Risk & Radiation Free - Could be integrated in routine TTE Exam with no extra hardware requirement - TTE Machines widely available - Offers true transmural mapping to identify challenging SoO that may require epicardial access or advanced ablation technology (e.g. ethanol, bipolar ablation) | | | - Non-invasive high density simultaneous activation mapping covering the full heart surface - Offers single-beat mapping of isolated, rare and/or multi-focal arrhythmias - Easy to use & apply to all patient sizes - Data processing quick and allows for real time intraprocedural beat mapping - Long-standing experience and numerous validation studies in-vivo in humans for ventricular mapping | |
| Disadvantages | - Current acquisition workflow not practical for low burden VE and/or multifocal VEs - 2D Acquisition - interplanar gaps and risks to miss true SoO - Echo-quality – limitations in patients e.g. with severe obesity and lung disease, metallic prosthesis causing artefacts etc - Dedicated research ultrasound & custom-made postprocessing software required - Data processing time consuming & high manual input - Extensive Scarring may prevent accurate mapping | | | - Depending on source model, only epicardial potentials and no reliable information about site of origin on transmural axis available - Special equipment required - Costly (single use vest, advanced imaging) - CT scan with radiation exposure (alternative: CMR-ECGI or imageless ECGi proposed) - Susceptible to noise/electromagnetic interference in hospital environment - Does not represent septal segments and intracavitary structures | |
| Future Work & Next Steps | - Automation of data processing - Full 3D Single Beat Acquisition - Retrospective Acquisition - Integration in Clinical Echocardiogram - Real-Time Intraprocedural Use (TOE/ICE) | | | - 4D CT or CMR for dynamic registration across cardiac cycle and cardiac-phase specific EGM reconstruction / projection / visualisation - Improved algorithms for reliable and accurate transmural activation mapping - Integration and real time review of ECGI maps in electro-anatomical mapping systems | |
